# Long Non-coding RNA and mRNA Profile of Liver Tissue During Four Developmental Stages in the Chicken

**DOI:** 10.3389/fgene.2020.00574

**Published:** 2020-06-16

**Authors:** Chunyou Ning, Tianyuan Ma, Silu Hu, Zhongxian Xu, Pu Zhang, Xiaoling Zhao, Yan Wang, Huadong Yin, Yaodong Hu, Xiaolan Fan, Bo Zeng, Mingyao Yang, Deying Yang, Qingyong Ni, Yan Li, Mingwang Zhang, Huailiang Xu, Yongfang Yao, Qing Zhu, Diyan Li

**Affiliations:** ^1^Farm Animal Genetic Resources Exploration and Innovation Key Laboratory of Sichuan Province, College of Animal Science and Technology, Sichuan Agricultural University, Chengdu, China; ^2^College of Life Science, Sichuan Agricultural University, Ya’an, China

**Keywords:** long non-coding RNA, mRNA, liver, chicken, development

## Abstract

The liver is the major organ of lipid biosynthesis in the chicken. In laying hens, the liver synthesizes most of the yolk precursors and transports them to developing follicles to produce eggs. However, a systematic investigation of the long non-coding RNA (lncRNA) and mRNA transcriptome in liver across developmental stages is needed. Here, we constructed 12 RNA libraries from liver tissue during four developmental stages: juvenile (day 60), sexual maturity (day 133), peak laying (day 220), and broodiness (day 400). A total of 16,930 putative lncRNAs and 18,260 mRNAs were identified. More than half (53.70%) of the lncRNAs were intergenic lncRNAs. The temporal expression pattern showed that lncRNAs were more restricted than mRNAs. We identified numerous differentially expressed lncRNAs and mRNAs by pairwise comparison between the four developmental stages and found that *VTG2*, *RBP*, and a novel protein-coding gene were differentially expressed in all stages. Time-series analysis showed that the modules with upregulated genes were involved in lipid metabolism processes. Co-expression networks suggested functional relatedness between mRNAs and lncRNAs; the DE-lncRNAs were mainly involved in lipid biosynthesis and metabolism processes. We showed that the liver transcriptome varies across different developmental stages. Our results improve our understanding of the molecular mechanisms underlying liver development in chickens.

## Introduction

The liver is the largest metabolic organ, and it is involved in the metabolism of numerous biologically important compounds, synthesis of essential proteins, detoxification of exogenous materials, and host defense against invading pathogens (Grant, 1991; Brockmöller and Ivar, 1994; Calne, 2000; Rui, 2014). The liver produces a large number of fatty acids and play a crucial role in lipid transportation through lipoprotein synthesis, depending on species (Nguyen et al., 2008; Mashek, 2013). In chickens, over 70% of the *de novo* lipogenesis occurs in the liver (Leveille et al., 1968; O’Hea and Leveille, 1969), especially during the egg production period. The production of egg yolk proteins in chicken liver is mainly regulated by several steroid hormones, such as estrogen. As hens mature sexually, the secretion of hydrophobic lipids including free fatty acids, triacylglycerols, and cholesteryl esters begins in the liver and these are assembled together to form two major components of yolk protein precursors including very low density apolipoprotein II (ApoVLDL II) and vitellogenin II (VTG II) under estrogen stimulation (Wiskocil et al., 1980; Denslow et al., 1999; Walzem et al., 1999). These particles are subsequently transported to blood and circulated to the developing oocyte for embryo growth and development (Nimpf and Schneider, 1991; Schneider, 1995).

Large-scale transcriptome analyses reveal that about 75% of the human genome can be transcribed into RNAs, and most of these transcripts are defined as different types of non-coding RNAs (ncRNAs) (Djebali et al., 2012; Liu et al., 2013). Of these, long non-coding RNAs (lncRNAs) are the most abundant and play versatile role in gene regulation of expression (Derrien et al., 2012a). The lncRNAs are generally >200 nucleotides (nt) in length with little or no coding potential (Esteller, 2011). LncRNAs were initially considered to be “noise” of genome transcription and to lack biological function. However, studies in different species have fully demonstrated that lncRNAs are an important regulatory factor in a series of biological processes, such as controlled recruitment of transcription factors (Martianov et al., 2007; Maamar et al., 2013; Ng et al., 2013), regulation of mRNA decay and translation rate (Gong and Maquat, 2011; Carrieri et al., 2012), and formation and function of subnuclear structures (Mao et al., 2010; Yang et al., 2011).

The chicken is an established model organism for studying vertebrate development (Stern, 2005). Compared with mammals, the liver is the accessory organ of lipid biosynthesis in the chicken (Leveille et al., 1975). Thus, liver is responsible for lipid metabolism and homeostasis that play a critical effect on chicken growth and laying performance. It is also important for birds to adapt environmental changes through the regulation of the lipid metabolism in liver (Bedu et al., 2002). Although the genes and gene products related to hepatic lipid metabolism in chicken liver are very similar to those in mammals, they differed in specialized functions (Kirchgessner et al., 1987). For example, the microsomal triglyceride transfer protein (MTTP) is known to assist lipoprotein assembly to form very low density lipoprotein (VLDL) in mammals (Hussain et al., 2008, 2012); however, a previous study indicated that upregulation of MTTP in the liver does not require increased VLDL assembly during laying process in chicken (Ivessa et al., 2013). In addition, a battery of genes associated to lipid metabolism were lost from the chicken genome during the evolutionary process (Ðaković et al., 2014). Most RNA-seq studies in chickens have focused on protein-coding genes and ignored lncRNAs because of their poor conservation and low expression abundance in tissues compared with mRNAs. Therefore, the repertoire of potential genes and lncRNAs involved in hepatic lipid metabolism remains to be completely elucidated in laying chickens.

There, in this study, we comprehensively investigated the lncRNAs and mRNAs in chicken liver at four developmental stages using high-throughput RNA sequencing technology. We identified many differentially expressed transcripts and explored these enrichment pathways across the four stages. In addition, by constructing a co-expression network of lncRNA and mRNAs, we predicted the potential functions of lncRNAs. These results would contribute to better comprehension of transcriptome changes and comparative understanding of molecular mechanisms of liver development in chickens.

## Materials and Methods

### Animals and Liver Tissue Collection

In this study, the healthy hen chickens of Luhua (a Chinese indigenous breed) were obtained from the Experimental Chicken Farm of Sichuan Agricultural University. Chickens were examined at the four developmental stages: juvenile period (JP, 60 days of age), sexual maturity period (SM, 133 days of age), peak laying period (PL, 220 days of age), and broodiness period (BP, 400 days of age). Birds were euthanized by intravenous injection containing 2% pentobarbital sodium (25 mg/kg of BW). Liver tissue was rapidly collected from three biological replicates at each stage and promptly frozen in liquid nitrogen. All samples were stored at −80°C until total RNA extraction. All experimental methods and sample collection of this study were conducted in accordance with guidelines provided by the Institutional Animal Care and Use Committee (IACUC) of Sichuan Agricultural University, under permit No. DKY-B20171910.

### RNA Isolation, Library Preparation, and Sequencing

Total RNA of each sample was extracted using RNAiso Plus reagent (TaKaRa, Otsu, Shiga, Japan) following the manufacturer’s instruction. We estimated the integrity and quality of total RNA using Bioanalyzer 2100 system (Agilent Technologies, Palo Alto, CA, United States) with an RNA 6000 Nano kit. Subsequently, 12 strand-specific libraries were generated after depleting rRNA by the Ribo-Zero^TM^ Gold Kit (Illumina, San Diego, CA, United States) and then sequenced on the Illumina HiSeq X Ten platform (Illumina Inc., San Diego, CA, United States) with a paired-end sequencing length of 150 bp (PE150) at Novogene Corporation (Beijing, China).

### Identification of lncRNAs

We removed the low-quality reads, adaptor sequences, empty reads, and ribosomal (r)RNA reads from the raw data in order to obtain high-quality lncRNAs. The processed reads were mapped to the chicken reference genome (*Gallus gallus-5.0*) using HISAT2 2.1.0 (Kim et al., 2015), and the mapped reads were assembled by Stringtie v1.3.3 (Pertea et al., 2015). Then, we merged the assembled transcripts into consensus transcripts using custom Python scripts after filtering reads with length less than 200 nt. Coffcompare v2.2.1 was applied to removed transcripts annotated as “C” and “=” (“C” for partial match, and “=” for full match) (Trapnell et al., 2010). Next, the remaining known protein-coding transcripts were removed by BLASTX and Hmmscan (Eddy, 2009). The Coding Potential Calculator (CPC) (Kong et al., 2007) was used to assess the coding potential of the remaining transcripts; transcripts with FPKM > 0 (where FPKM = fragments per kilobase of transcript per million mapped reads) in at least one biological replicate were annotated as lncRNAs. The pipeline of lncRNA identification is depicted in Supplementary Figure S1.

### Classification of lncRNAs

The identified lncRNAs were classified by FEELnc (Wucher et al., 2017), a tool for lncRNA prediction and annotation. According to the location with corresponding gene, lncRNAs were classified into two categories: intergenic lncRNAs and intragenic lncRNAs. The intragenic lncRNAs can be further subclassified into four categories: (1) sense intronic lncRNAs, (2) antisense intronic lncRNAs, (3) sense exonic lncRNAs, and (4) antisense exonic lncRNAs.

### Transcript Expression Level Analysis

Transcript abundance was evaluated by FPKM values of mRNAs and lncRNAs using StringTie v1.3.3 (Pertea et al., 2015). The transcripts that exhibited FPKM > 0.1 were used to filter the expressed protein-coding genes. Subsequently, log2-transformed values of FPKM + 1 were used for further analysis. For reliability of the experimental data, Spearman correlations were checked across the four developmental stages. *t*-Distributed stochastic neighbor embedding (*t*-SNE) was performed using R software and hierarchical clustering was carried out using MultiExperiment Viewer (MeV v4.9.0) (Howe et al., 2010). The differentially expressed genes (DEGs) and lncRNAs transcripts (DE-lncRNAs) were identified using edgeR package^1^ based on the read count data (Robinson et al., 2009). The significant DEGs and DE-lncRNAs were screened with a false discovery rate <0.05 and | fold change (FC)| ≥ 1.5 as cutoff.

### Time-Series Analysis

We performed the four time-series analysis using the Short Time-series Expression Miner (STEM) (Ernst and Bar-Joseph, 2006). Different colors represented significantly enriched model profiles (Bonferroni-adjusted *P-*values ≤ 0.05), where model profiles with the same color were considered the same cluster of expression profiles.

### LncRNA-mRNA Co-expression Network Analysis

To predict the function of lncRNAs, a co-expression network was constructed by Weighted Gene Co-expression Network Analysis (WGCNA) in the R package (Langfelder and Horvath, 2008). We calculated the co-expression network based on the DEGs and DE-lncRNAs that were screened by edgeR. The “co-expression modules” detected by average linkage hierarchical clustering represented genes with coordinated expression patterns.

### Functional Enrichment Analysis for Genes

The DEGs were subjected to functional enrichment analysis for Gene Ontology (GO) terms and Kyoto Encyclopedia of Genes and Genomes (KEGG) pathway categories using Metascape (Zhou et al., 2019) online tool^2^. The GO terms and KEGG pathways with *P*-values < 0.01 were considered significant.

### Quantitative Real-Time PCR Validation

To verify the reliability of the expression profiles of RNA-sequencing data, we randomly evaluated expression of five each for DEGs and DE-lncRNAs by quantitative real-time PCR (qPCR). The primers used for qPCR are shown in Supplementary Table S1. The cDNA was synthesized using the EasyScript One-Step gDNA Removal and cDNA Synthesis SuperMix (Transgen Biotech, Beijing, China) following the manufacturer’s recommendation. We performed qPCR on a CFX96 Real-Time PCR Detection system (Bio-Rad Laboratories, Hercules, CA, United States) using TransStart Top Green qPCR SuperMix (Transgen Biotech, Beijing, China). The reaction volume was 20 μl, containing 1 μl of template cDNA, 0.4 μl of forward and reverse primers, 10 μl of Top Green qPCR SuperMix, and 8.2 μl of RNase-free water. The qPCR measurements were evaluated in triplicate followed the qPCR system: 94°C for 30 s, followed by 40 cycles of 94°C for 5 s and 30 s at the Tm indicated in Supplementary Table S1. The melting curve analysis was carried out from 65 to 95°C with increments of 0.5°C for 5 s. The expression levels of each transcript were normalized to that of *ACTB* used as endogenous control. The fold change in expression levels was determined using the 2^–ΔΔCt^ method.

## Results

### Data Summary of lncRNAs and mRNAs Profile in Chicken Liver

To systematically explore the hepatic transcriptome during development in chicken, we constructed 12 cDNA libraries from 12 female Luhua chickens in four crucial stages during development: JP (day 60), SM (day 133), PL (day 220), and BP (day 400). The strand-specific libraries were sequenced on Illumina’s HiSeq platform. A total of 195.53 Gb of raw data with 150-bp paired-end sequences were generated. After filtering adaptors and low-quality reads, 192.31 Gb clean reads were retained corresponding to an average of ∼16.03 Gb of high-quality reads per sample. The high-quality reads were mapped to the chicken genome (*G. gallus-5.0*) with a mapping rate of 92.20 to 96.26% using HISAT2.1.0 (Supplementary Table S2).

### Genomic Characterization and Classification of lncRNAs

**FIGURE 1:**
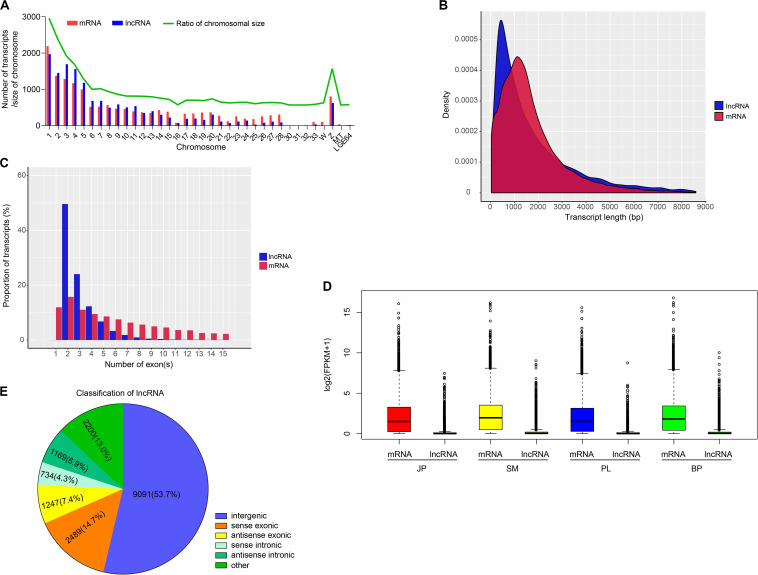
Genomic characterization of lncRNAs and mRNAs. **(A)** Chromosome distribution of lncRNAs and mRNAs identified in chicken liver. **(B)** The distribution of transcript length of lncRNAs and mRNAs. **(C)** The proportion of transcripts with different number of exons. **(D)** The expression level of mRNAs and lncRNAs in four developmental stages. **(E)** The classification of lncRNAs.

To identify putative lncRNAs in chicken liver, we analyzed the homology of transcripts with known genes, inclusion of a known protein-coding region, and their coding potential. As a result, we obtained 16,930 putative lncRNAs that were expressed in at least one biological replicate (FPKM > 0), and 18,260 mRNAs that were substantially expressed (FPKM > 0.1 in at least one replicate) (Supplementary Tables S3, S4). We found that 14,959 (88.36%) lncRNAs and 15,983 (87.53%) mRNAs were assembled on chromosomes of chicken (Figure 1A and Supplementary Table S5), and most of these transcripts tended to be distributed in the large chromosomes (nos. 1–5 and the Z chromosome). Additionally, the numbers of mRNAs and lncRNAs were highly correlated with chromosome size (*r* = 0.9712 and 0.9371, respectively) (Supplementary Figure S2). Further comparative analysis showed that lncRNAs were shorter with fewer exons and were expressed at a lower level than that of mRNAs (Figures 1B–D).

According to their genomic location, we used FEELnc to separate the 14,730 identified lncRNAs into five groups: 9091 intergenic lncRNAs (lincRNAs, 53.7%), 2489 sense exonic overlapping lncRNAs (14.7%), 1247 antisense exonic overlapping lncRNAs (7.4%), 734 sense intronic overlapping lncRNAs (4.3%), and 1169 antisense intronic overlapping lncRNAs (6.9%) (Figure 1E). The results of functional enrichment showed that genes whose exons and introns overlapped with lncRNAs with the most significantly enriched GO categories were annotated with terms that involved development and cell activities, such as regulation of system process (GO:0044057), developmental growth (GO:0048589), regulation of cell adhesion (GO:0030155), and cell surface receptor signaling pathway involved in cell–cell signaling (GO:1905114). KEGG pathways were enriched with terms such as MAPK signaling pathway (hsa04010), endocytosis (hsa04144), and prolactin signaling pathway (hsa04917) (Supplementary Table S6). These results indicated that the potential functions of exon- and intron-overlapping lncRNAs may be closely involved in these enriched categories and pathways.

### Expression Profiles of mRNAs and lncRNAs

**FIGURE 2:**
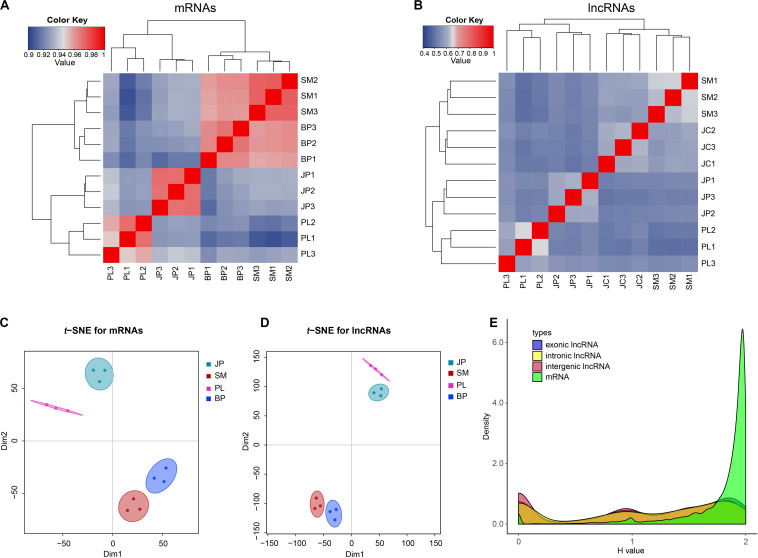
The expression profiles of mRNAs and lncRNAs. **(A,B)** Dynamic changes in expression profile of mRNAs and lncRNAs. The top and left panel is the sample tree and the value represents the pairwise Spearman correlation. **(C,D)** Two-way *t*-SNE plot of mRNAs and lncRNAs based on expression profiles. **(E)** Distributions of Shannon entropy-based temporal specificity scores were calculated for distinct types of lncRNAs and mRNAs.

Based on the expression change of mRNAs and lncRNAs, we calculated Spearman correlations between each pair of samples. The results showed that both the mRNAs and lncRNAs of 12 samples were grouped into four clusters based on their developmental stages: samples at the PL and JP stages were separated from the SM and BP stages, whereas the SM and BP stages were more convergent than the PL and JP stages (Figures 2A,B). Similarly, *t*-SNE plots of 12 samples indicated that mRNAs and lncRNAs were expressed in a stage-specific manner (Figures 2C,D). In addition, we found that the correlation between two consecutive stages of lncRNAs was weaker than that for the mRNAs (Figures 2A,B). This observation indicates that lncRNAs have a narrower time windows on expression profiles and more restricted temporal expression than mRNAs. To further validate the inference, we used the Shannon entropy (*H*) value to calculate the specificity of expression profile across developmental stages. Compared with mRNAs, all three types of lncRNAs, intergenic, exon-overlapping, and intron-overlapping, showed increased temporal specificity (Figure 2E).

### Identification of Differentially Expressed mRNAs and lncRNAs

To identify the DEGs and DE-lncRNAs in chicken liver, we performed pairwise comparisons of expression level between the four developmental stages with a threshold of |FC| > 1.5 and a false discovery rate < 0.05. Overall, by comparing six pairs, we have identified 4405 unique mRNAs and 752 unique lncRNAs, which were significantly differentially expressed over four developmental stages. A greater number of DEGs were obtained from JP versus SM, followed by JP versus BP and SM versus PL, whereas most DE lncRNAs were detected at SM versus PL, followed by JP versus SM and JP versus BP (Supplementary Tables S7, S8). The results of functional enrichment showed that most of these DEGs were enriched in metabolic processes, hormone stimulus, and developmental processes, such as fatty acid metabolic process (GO:0006631), oxidoreductase activity (GO:0016491), regulation of hormone levels (GO:0010817), and reproductive structure development (GO:0048608) (Supplementary Table S9).

**FIGURE 3:**
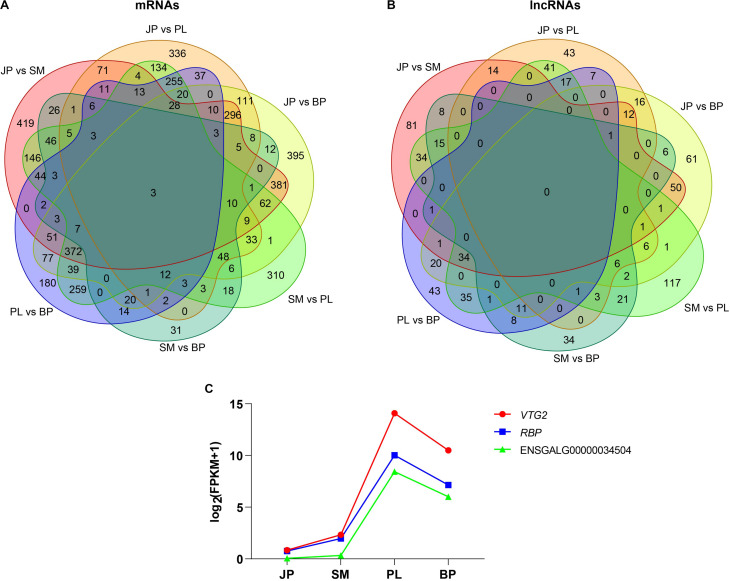
Differentially expressed mRNAs and lncRNAs. The Venn diagrams of common differentially expressed mRNAs **(A)** and lncRNAs **(B)** in four developmental stages. **(C)** Dynamic expression profiles of *VTG2*, *RBP*, and a novel gene (ENSGALG00000034504).

Subsequently, we analyzed the common DEGs and DE lncRNAs for the four developmental stages of chicken liver. The Venn diagram showed that only three genes were differentially expressed in all four developmental stages, and no common DE lncRNAs were identified in these stages (Figures 3A,B). The three common DEGs were vitellogenin 2 (*VTG2*), riboflavin binding protein (*RBP*), and a novel gene (ENSEMBL_Id: ENSGALG00000034504). Interestingly, we found that all three protein-coding genes had the same pattern of expression; that is, expression increased during the first three developmental stages, peaked at the PL stage, and then declined at the BP stage (Figure 3C), indicating that these genes contributed to lipoprotein synthesis and egg yolk formation for meeting demand for egg production at the PL stage.

### Time-Series and Co-expression Network Analysis of mRNAs and lncRNAs

**FIGURE 4:**
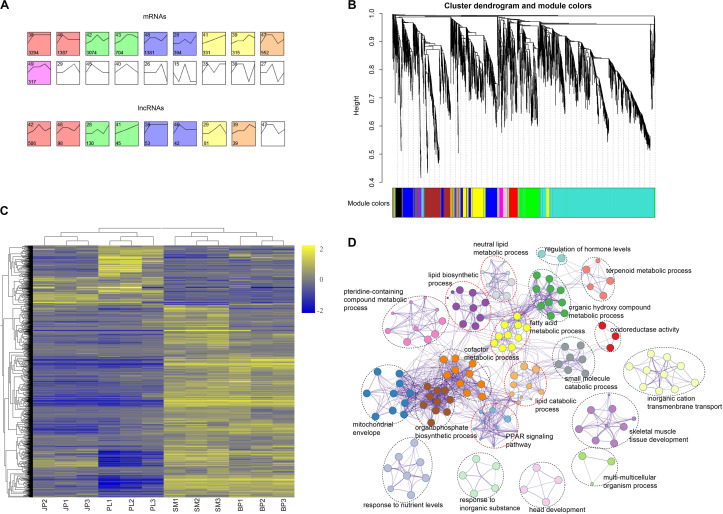
Time-series model and co-expression networks of lncRNAs and mRNAs. **(A)** Time-series modules of mRNAs and lncRNAs. The top panel shows mRNAs and the second panel shows lncRNAs. Numbers in the top left corner indicate module number. Numbers in the lower left corner indicate number of mRNAs or lncRNAs in each module. The same color was used to represent each cluster. **(B)** Co-expression network constructed by the WGCNA method. The top panel shows cluster dendrogram obtained by average linkage hierarchical clustering. The low panel shows co-expression modules of lncRNAs and mRNAs with different colors. **(C)** Heat map showing the mRNAs corresponding to the largest three co-expression network modules of lncRNAs. Values in heat map represent log2(FPKM + 1) of each mRNA in each sample minus the mean value of each mRNA across all samples. **(D)** Functional categories of the mRNAs corresponding to the largest three co-expression network modules of lncRNAs.

To further investigate the global expression pattern of mRNAs and lncRNAs during four hepatic developmental stages in chicken, we conducted a time-series analysis using STEM. A total of 11,749 mRNAs and 994 lncRNAs were partitioned into 6 and 5 module profiles, respectively, comprising 10 and 8 significantly enriched (Bonferroni-adjusted *P*-value ≤ 0.05) model profiles for mRNAs and lncRNAs, respectively (Figure 4A and Supplementary Tables S10, S11). In these model profiles, we found that mRNAs and lncRNAs existed in some model profiles with similar dynamic expression patterns, suggesting that their functions were highly correlated. Subsequently, we used the mRNAs in the yellow module (monotonic trajectory with increased expression pattern across time) to analyze the functional distribution. Interestingly, the genes in the yellow module were mainly enriched in sulfur compound metabolic process (GO:0006790), lipid biosynthetic process (GO:0008610), cellular response to hormone stimulus (GO:0032870), and glucose homeostasis (GO:0042593) (Supplementary Table S12). This finding was consistent with the biological function of chicken liver in different periods.

Due to the characteristics of lncRNA and the lack of annotation libraries, it remains difficult to predict the function of lncRNAs. Here, we explored functional relatedness between mRNAs and lncRNAs using co-expression analysis. We built the co-expression network based on a non-redundant list of DEGs and DE-lncRNAs by WGCNA. Ultimately, 14 co-expression modules of lncRNAs and mRNAs were identified (Figure 4B). To predict the function of the DE-lncRNAs, we only considered the top three modules of lncRNA and these overlapping genes (Figure 4C and Supplementary Table S13), which accounted for 63.22% of the total genes in the 14 modules. We submitted these genes to Metascape for functional enrichment and the result showed that the co-expression genes in the top three modules were mainly enriched in terms related to lipids (Figure 4D and Supplementary Table S14), such as lipid biosynthetic process (GO:0008610), neutral lipid metabolic process (GO:0006638), fatty acid metabolic process (GO:0006631), and lipid catabolic process (GO:0016042). The KEGG analysis of the peroxisome proliferator-activated receptor (PPAR) signaling pathway (hsa03320) was also closely related to intracellular synthesis of lipid. These results indicated that most of the DE-lncRNAs may be involved in lipid synthesis and metabolism in the liver of chicken.

### Quantitative Real-Time PCR Validation of Gene Expression

**FIGURE 5:**
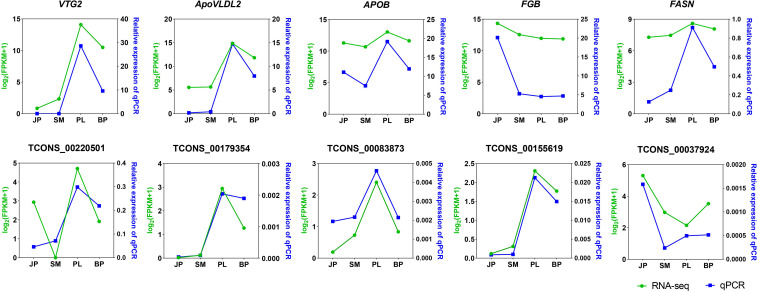
Validation of five expressed DEGs and DE lncRNAs by qPCR. The *x*-axis represents the four developmental stages. The *y*-axis indicates the relative expression of each gene; green lines are log2(FPKM + 1) values of RNA-seq and the blue lines show relative expression by qPCR.

We selected five DEGs (*VTG2*, *ApoVLDL2*, *APOB*, *FGB*, and *FASN*) and five DE-lncRNAs (TCONS_00220501, TCONS_00179354, TCONS_00083873, TCONS_00155619, and TCONS_00037924) for validation by qPCR (Figure 5). The relative expression of the five DEGs and five DE lncRNA of each sample by qPCR was compared with the transformed log2(FPKM + 1) values of RNA-seq. All genes except *FGB* showed the same expression pattern in both qPCR and RNA-seq, having the highest expression level at the PL stage. The altered expression pattern of DEGs and DE-lncRNAs at most stages were also consistent with the RNA-seq data. These results illustrated the reliability of our RNA-seq data and confirmed the accuracy of the identified transcripts.

## Discussion

It is well established that the liver is the major organ of lipid biosynthesis in the chicken (Leveille et al., 1968; O’Hea and Leveille, 1968, 1969). In laying hens, the liver synthesizes a series of lipoproteins and most of the additional minor yolk precursors. This biological process represents a complex and highly orchestrated program of gene expression and regulation of various regulatory molecules, such as microRNAs (miRNAs) and lncRNAs. Although the mRNA and miRNA transcriptome in chicken liver has been partially explored in previous studies (Wang et al., 2014; Li et al., 2015, 2016), a systematic evaluation of the lncRNA repertoire across chicken liver developmental stages is lacking. To further investigate change in the transcriptome expression profile in different developmental stages, we performed RNA-seq and identified lncRNAs and mRNAs at days 60, 133, 220, and 400, representing four developmental stages of the chicken. In total, we obtained 192.31 Gb of clean data from 12 libraries and identified 16,930 lncRNAs and 18,260 mRNAs. We found that lncRNAs had several specific genomic characteristics compared with mRNAs (shorter sequences, fewer exons, lower expression level, and more temporal specificity) consistent with results in other studies (Derrien et al., 2012b; Peng et al., 2014; Muret et al., 2017; Jin et al., 2018).

According to our results, lncRNAs and mRNAs were expressed in a stage-dependent manner of expression profiles. Spearman correlation and *t*-SNE showed that the SM stage is more closed to BP stage, which means lncRNA and mRNA had similar expression profiles at the two stages. Indeed, the capacity of hepatic lipogenesis weakens because of the absence of egg production in the SM and BP stages, which is in line with the lipogenic enzyme activities involved in this process (Pearce, 1971). We found that lncRNAs were expressed for shorter duration compared with mRNAs, suggesting that lncRNAs have more restricted temporal expression than mRNAs (Pauli et al., 2012).

Pairwise comparisons of lncRNAs and mRNAs were performed to identify DEGs and DE-lncRNAs. We found that most of these DEGs were enriched in various lipid metabolic and developmental processes, especially between JP and PL stage, which were consistent with a study of Li et al. (2015). In addition, only three genes, *VTG2*, *RBP*, and a novel gene (ENSEMBL_Id: ENSGALG00000034504), were found to be differentially expressed in all four stages. These three DEGs had similar expression profiles, reaching peak expression at the PL stage, indicating their important roles involved in chicken liver development and egg production. It is clear that *VTG2*, a marker gene in yolk precursor formation, has a vital role in follicle development and maturation (Schneider, 1996). The *VTG2* gene encodes vitellogenin, which is dependent on estrogen stimulation (Denslow et al., 1999; Li et al., 2014). In laying hens, three subtypes of VTG are identified in liver; the ratio of VTG I:II:III RNA is 4:10:1 (Evans et al., 1988). As a glycophospholipoprotein, VTG is synthesized in the liver and then deposited in the rapidly developing oocytes. It has a critical effect on reproduction performance by providing nutrients and functional substances for embryonic development (Richards, 1997). The protein encoded by *RBP*, which is stored in eggs, is also essential for maintaining growth and developing embryos. It binds riboflavin (vitamin B2) and is involved in its transport from the serum to the oocyte (White and Merrill, 1988). Changes in the expression levels of *VTG2* and *RBP* in our study were consistent with the biological functions of chicken liver in different periods. Surprisingly, the two egg-expressed genes, *VTG2* and *RBP*, were lost in mammals during evolution (Tian et al., 2010). Unlike the oviparous vertebrates that depend on nutritional reserves, mammals have evolved placenta to nourish their embryos and mammary gland as milk resources for early offspring (Brawand et al., 2008; Tian et al., 2010). In addition, we identified a novel protein-coding gene that was differentially expressed in liver at all four developmental stages, indicating that it may participate in lipid metabolism in chicken liver.

Time-series analysis suggested that both lncRNAs and mRNAs were dynamically expressed in liver during four developmental stages and have similar expression patterns. Genes in the yellow module with upregulated expression, such as *ACACA* (Cronan and Waldrop, 2002), *CYP3A4* (Wang et al., 2008), and *INSIG1* (Gong et al., 2006), were involved in metabolic activity and lipid homeostasis. At the PL stage, the liver is an important organ for the synthesis of egg yolk substances, and many genes related to lipid metabolism and synthesis of important yolk proteins are highly expressed to meet the demand for egg production. Besides, we found that some genes maintained a high expression level at the BP stage in the yellow module, such as *PRLR*, which was enriched in hormone stimulus-related process. The *PRLR* gene, which encodes prolactin receptor and modulates prolactin activity in an endocrine and autocrine manner, is generally accepted as a candidate gene for the onset and maintenance of broodiness in birds (Wilkanowska et al., 2014). Studies also have illustrated that this gene polymorphisms are associated with egg production, body weight at hatch, and age at sexual maturity (Rashidi et al., 2012; Zhang et al., 2012). The function of these genes can explain the changes in expression levels during corresponding stages. To determine the potential function of lncRNAs, we constructed co-expression networks between DE-lncRNAs and DEGs using WGCNA. The co-expression networks can be applied to associate lncRNAs with functionally annotated mRNAs (van Dam et al., 2017). Transcripts with similar expression patterns are often biologically related and can therefore be divided into the same module by WGCNA. Our results showed that five clusters of GO and KEGG terms, including lipid biosynthetic process, neutral lipid metabolic process, fatty acid metabolic process, lipid catabolic process, and PPAR signaling pathway were enriched in the co-expressed genes. For example, *APOA1* encodes key components modulating lipid metabolism (Sorci-Thomas et al., 1989). Apolipoprotein A1 is the major protein of high-density lipoprotein (HDL), and it functions to regulate the reverse cholesterol transport (Segrest et al., 2000). The *SCD* gene encodes stearoyl CoA desaturase-1, which is located in endoplasmic reticulum membrane and transforms different saturated fatty acids into monounsaturated fatty acids (Ntambi and Miyazaki, 2004). These co-expressed genes play crucial roles in lipid biosynthesis and metabolism, suggesting the biological function of DE-lncRNAs by regulating hepatic lipogenesis and liver development in chicken.

## Conclusion

In summary, we profiled a systematic lncRNA repertoire in liver across different developmental stages of the chicken. Our results indicated the potential roles of lncRNA in liver development based on co-expression network, but further research is need for function validation. Our study provides a high-quality resource for future transcriptomic studies and improves the comparative understanding of molecular mechanisms of liver development in chickens.

## Data Availability Statement

The datasets generated for this study can be found in the National Center for Biotechnology Information (NCBI) Gene Expression Omnibus (GEO) with accession number GSE138152.

## Ethics Statement

The animal study was reviewed and approved by the Institutional Animal Care and Use Committee (IACUC) of Sichuan Agricultural University.

## Author Contributions

CN, YY, QZ, MY, and DL designed the study and drafted the manuscript. CN, PZ, ZX, XF, BZ, and DY carried out the feeding trial and collected the liver tissue samples. TM, SH, XZ, YW, HY, and YH extracted the liver tissue RNA and contributed to the data analysis. QN, YL, and MZ contributed to the qPCR validation. HX, QZ, and DL reviewed and improved this manuscript. All authors contributed to the manuscript and approved the submitted version.

## Conflict of Interest

The authors declare that the research was conducted in the absence of any commercial or financial relationships that could be construed as a potential conflict of interest.

## Footnotes

1 http://www.r-project.org/

2 http://metascape.org/gp/index.html#/main/step1

## Supplementary Material

FIGURE S1 Identification pipeline of lncRNAs.

FIGURE S2 Correlation between size of chromosome and number of identified transcripts: **(A)** mRNA and **(B)** lncRNAs.

FIGURE S3 The number of differentially expressed genes (DEGs, **A**) and lncRNAs (DE lncRNAs, **B**) identified by pairwise comparison.
