# Supplementary material for: Long Non-coding RNA and mRNA Profile of Liver Tissue During Four Developmental Stages in the Chicken
Source: Front Genet. 2020 Jun 16;11:574. doi: 10.3389/fgene.2020.00574 (PMC7309962; doi:10.3389/fgene.2020.00574)
Supplement: FIGURE S1 — Identification pipeline of lncRNAs. [file Data_Sheet_1.docx]

# Supplementary Figures and Tables

## Supplementary Figures


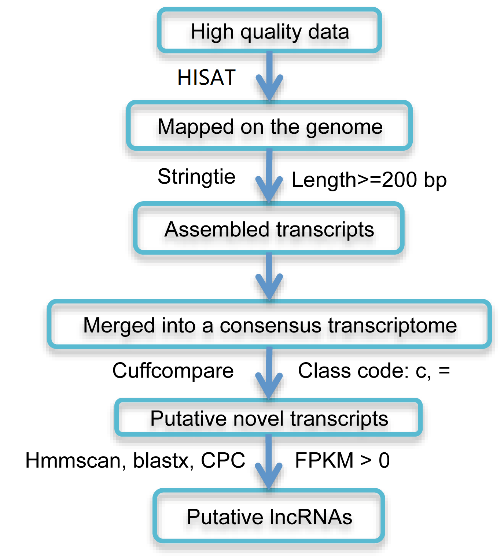


**Figure S1**. Identification pipeline of lncRNAs.


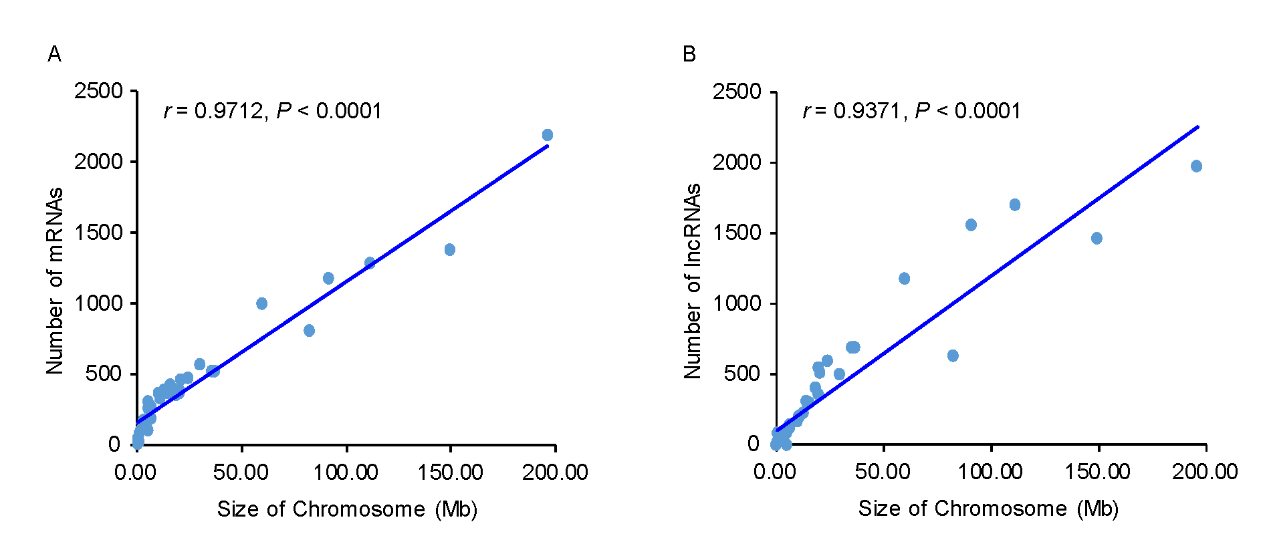


**Figure S2**. Correlation between size of chromosome and number of identified transcripts: (A) mRNA and (B) lncRNAs.


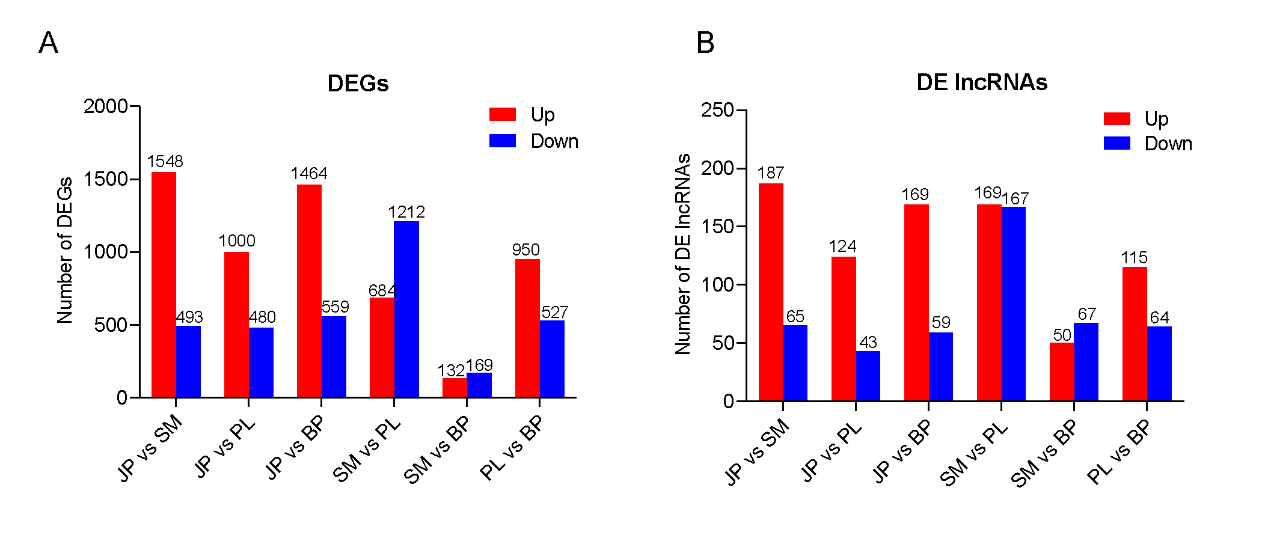


**Figure S3**. The number of differentially expressed genes (DEGs, A) and lncRNAs (DE lncRNAs, B) identified by pairwise comparison.

## Supplementary Tables

**Table S1.** Primer sequences of selected transcripts for qPCR.

**Table S2.** Data summary of samples for RNA sequencing.

**Table S3.** Identified lncRNAs in chicken liver.

**Table S4.** Identified mRNAs in chicken liver.

**Table S5.** Number of mRNAs and lncRNAs distributed on chromosome identified in chicken liver.

**Table S6.** Functional categories of genes overlapping with exonic and intronic lncRNAs.

**Table S7.** Differentially expressed mRNAs in pairwise stages.

**Table S8.** Differentially expressed lncRNAs in pairwise stages.

**Table S9.** Functional categories of differentially expressed mRNAs in pairwise stages.

**Table S10.** List of mRNAs in different module profiles of STEM.

**Table S11.** List of lncRNAs in different module profiles of STEM.

**Table S12.** Functional enrichment of genes in yellow module.

**Table S13.** List of top 3 modules of lncRNAs and related mRNAs.

**Table S14.** Functional categories of genes in co-expression network.
