# Supplementary material for: Long Non-coding RNA and mRNA Profile of Liver Tissue During Four Developmental Stages in the Chicken
Source: Front Genet. 2020 Jun 16;11:574. doi: 10.3389/fgene.2020.00574 (PMC7309962; doi:10.3389/fgene.2020.00574)
Supplement: Supplementary file 2 [file Data_Sheet_1.ZIP › Supplementary files/Table S2_data summary.docx]

**Table S2.** Data summary of samples for RNA sequencing

| **Sample description (age)** | **Sex** | **Sample ID** | **Raw reads number** | **Clean reads number** | **Clean reads radio (%)** | **Raw data (Gb)** | **Clean data (Gb)** | **Clean Q30 bases ratio (%)** | **Mapped ratio (%)** |
| --- | --- | --- | --- | --- | --- | --- | --- | --- | --- |
| Juvenile period (day 60) | Female | JP1 | 124,798,700 | 122,440,650 | 98.11 | 18.72 | 18.37 | 93.75 | 96.26 |
|  |  | JP2 | 132,577,412 | 129,311,850 | 97.54 | 19.89 | 19.40 | 93.25 | 95.43 |
|  |  | JP3 | 119,549,780 | 117,250,830 | 98.08 | 17.93 | 17.59 | 94.07 | 96.14 |
| Sexual maturity period (day 133) | Female | SM1 | 114,842,974 | 113,965,610 | 99.24 | 17.23 | 17.09 | 93.61 | 92.98 |
|  |  | SM2 | 102,220,456 | 101,444,286 | 99.24 | 15.33 | 15.22 | 93.50 | 92.64 |
|  |  | SM3 | 102,860,116 | 101,980,584 | 99.14 | 15.43 | 15.30 | 93.62 | 92.37 |
| Peak laying period (day 220) | Female | PL1 | 99,972,696 | 97,615,786 | 97.64 | 15.00 | 14.64 | 94.36 | 96.06 |
|  |  | PL2 | 116,546,254 | 114,085,474 | 97.89 | 17.48 | 17.11 | 94.71 | 95.69 |
|  |  | PL3 | 89,332,446 | 87,347,872 | 97.78 | 13.40 | 13.10 | 94.50 | 95.44 |
| Broodiness period（day 400） | Female | BP1 | 102,497,958 | 101,696,546 | 99.22 | 15.38 | 15.25 | 93.84 | 92.20 |
|  |  | BP2 | 94,653,538 | 93,114,962 | 98.37 | 14.20 | 13.97 | 94.00 | 92.36 |
|  |  | BP3 | 103,569,354 | 101,810,298 | 98.30 | 15.54 | 15.27 | 93.99 | 92.60 |
