# Supplementary figures and images for: Long Non-coding RNA and mRNA Profile of Liver Tissue During Four Developmental Stages in the Chicken
Source: Front Genet. 2020 Jun 16;11:574. doi: 10.3389/fgene.2020.00574 (PMC7309962; doi:10.3389/fgene.2020.00574)

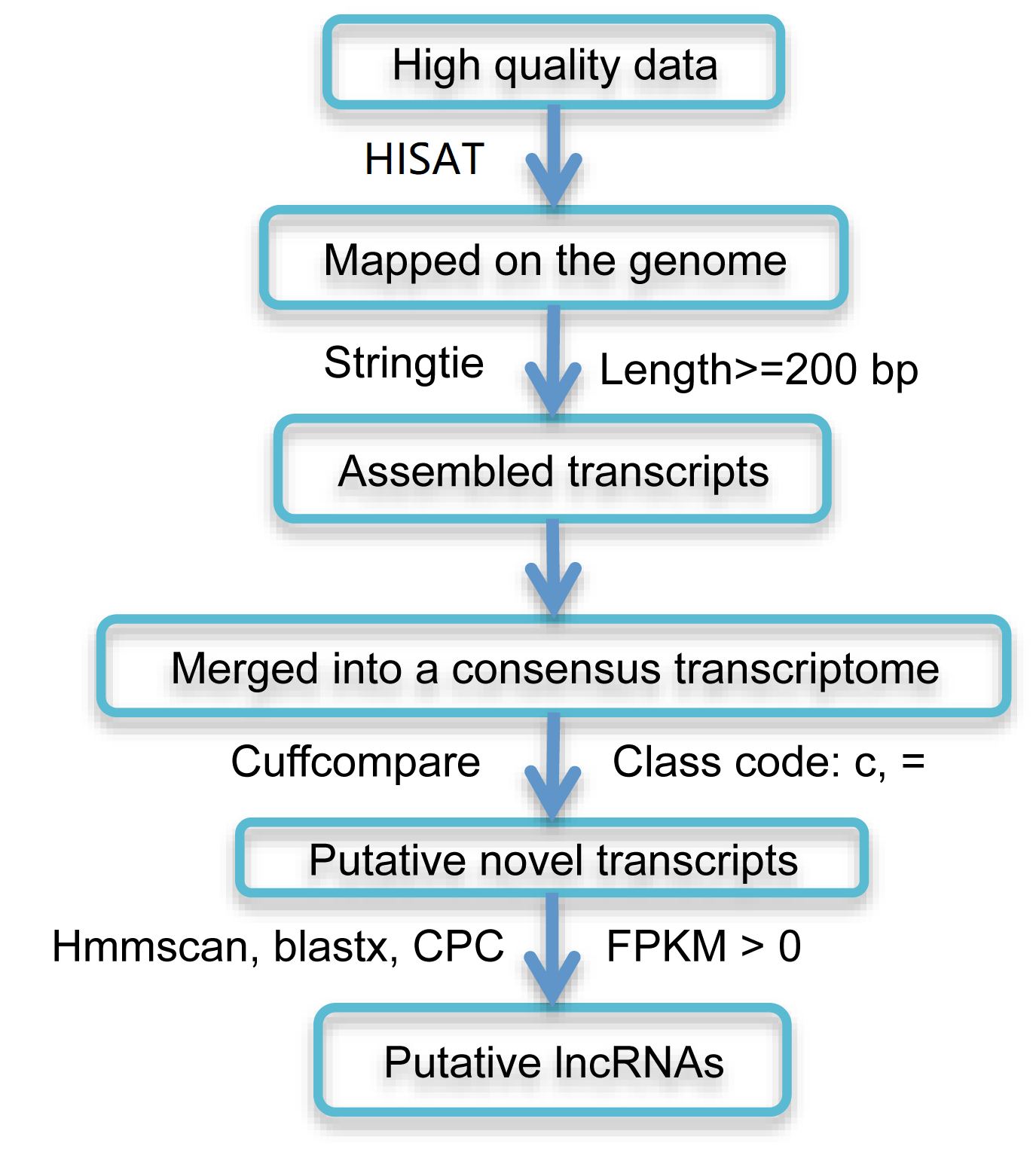

Supplement: Supplementary file 2 [file Data_Sheet_1.ZIP › Supplementary files/Figure S1.tif]

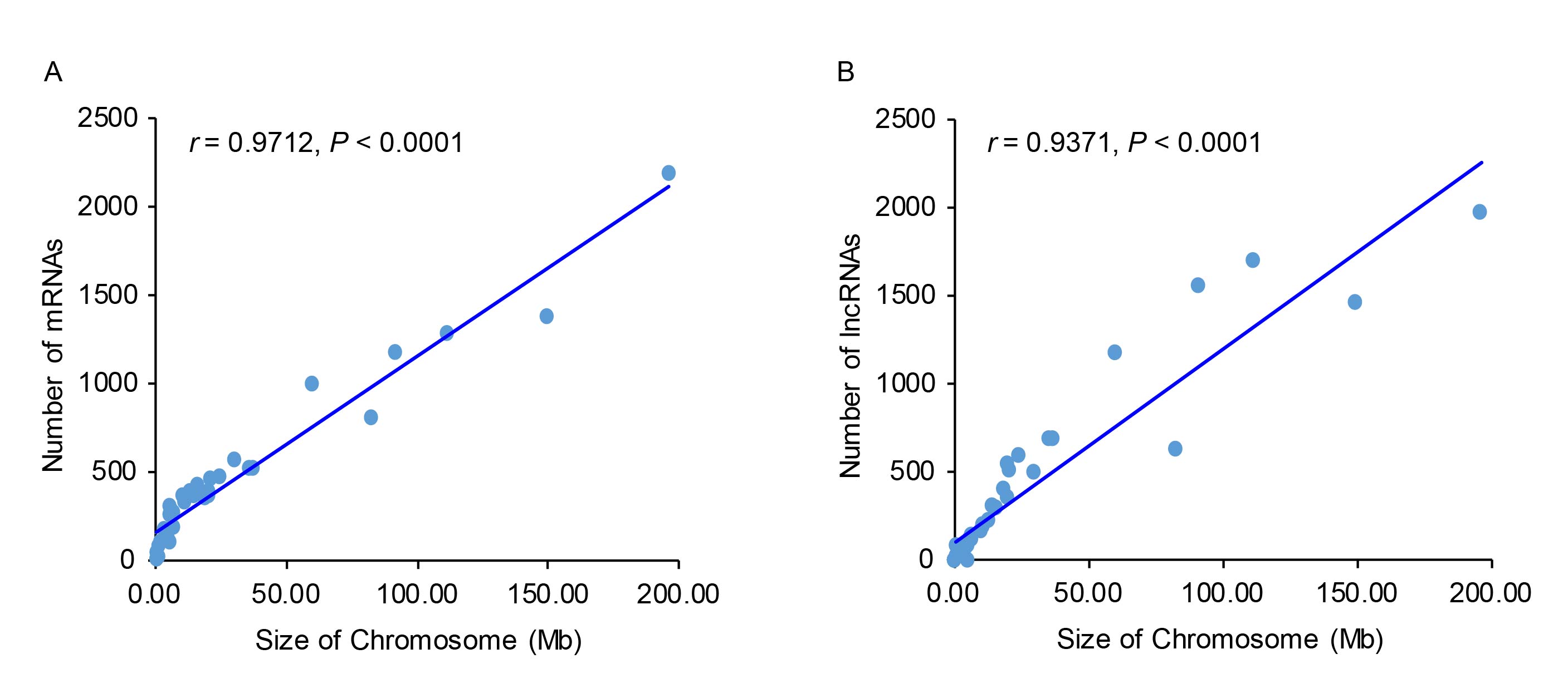

Supplement: Supplementary file 2 [file Data_Sheet_1.ZIP › Supplementary files/Figure S2.jpg]

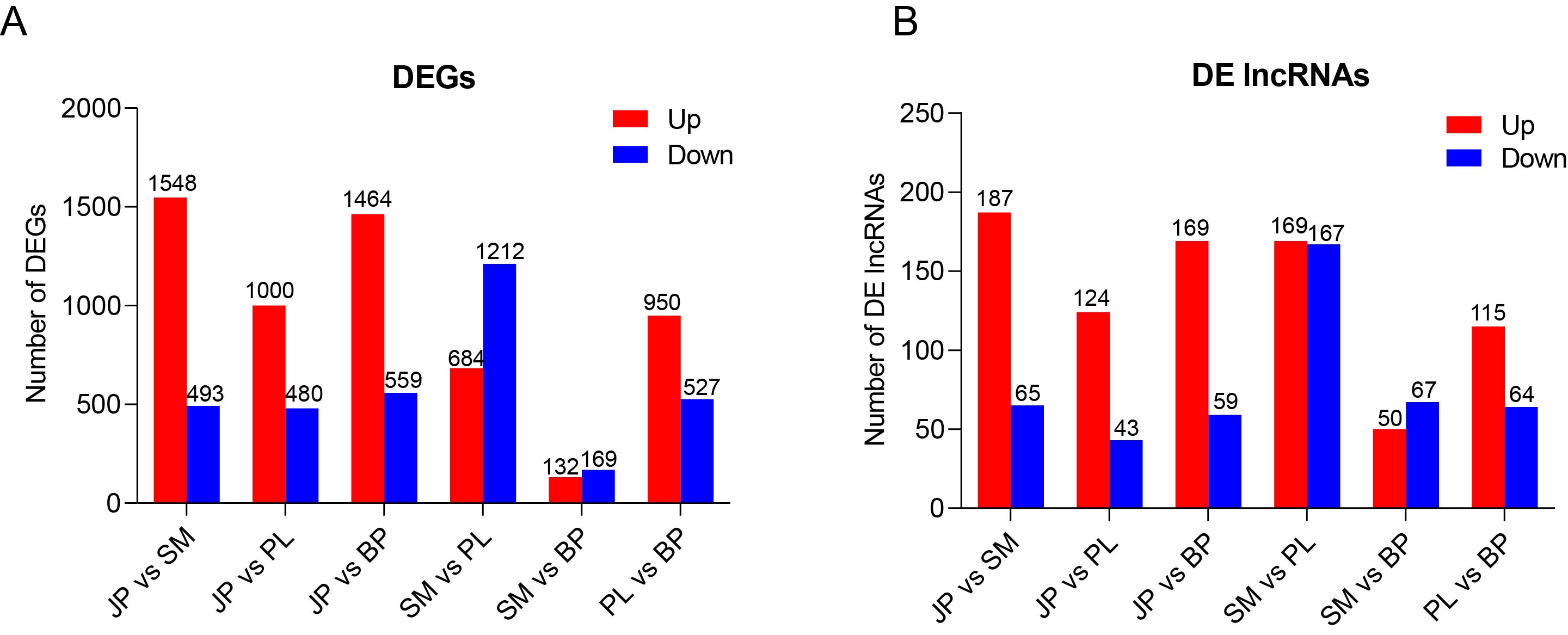

Supplement: Supplementary file 2 [file Data_Sheet_1.ZIP › Supplementary files/Figure S3.jpg]
